# Association of the Endothelial Activation and Stress Index (EASIX) with Clinical Outcomes in Patients with Pneumosepsis: A Retrospective Cohort Study

**DOI:** 10.3390/life16091429

**Published:** 2026-08-28

**Authors:** Derya Özyiğitoğlu, Ayşe Çapar, Emre Çapar, Şeyma Başlılar

**Affiliations:** 1 Department of Infectious Diseases and Clinical Microbiology, Sultan 2. Abdulhamid Han Training and Research Hospital, Istanbul 34668, Turkey; 2 Department of Anaesthesiology and Intensive Care Medicine, Sultan 2. Abdulhamid Han Training and Research Hospital, Istanbul 34668, Turkey; drayseyel@hotmail.com; 3 Department of Internal Medicine, Marmara University Pendik Training and Research Hospital, Istanbul 34899, Turkey; ecapar@yahoo.com; 4 Department of Chest Diseases, Sultan 2. Abdulhamid Han Training and Research Hospital, Istanbul 34668, Turkey; seymabaslilar@yahoo.com

**Keywords:** pneumosepsis, endothelial activation and stress index, endothelial dysfunction, mortality, intensive care unit

## Abstract

Background: Endothelial dysfunction is a key component of sepsis pathophysiology. The Endothelial Activation and Stress Index (EASIX) is a practical biomarker derived from routine laboratory parameters and indirectly reflects endothelial injury. This study aimed to evaluate the prognostic association of EASIX with in-hospital mortality and organ-support requirements among patients with pneumosepsis admitted to the intensive care unit (ICU). Methods: This retrospective cohort study included 1787 adult patients with pneumosepsis admitted to a tertiary ICU from January 2018 to December 2025. EASIX was calculated from laboratory parameters obtained at ICU admission, and log2-EASIX was used in all analyses. The primary outcome was in-hospital mortality. Secondary outcomes included the need for invasive mechanical ventilation (IMV), vasopressor therapy within the first 24 h, second-line vasopressor therapy, and continuous renal replacement therapy (CRRT) within the first 7 days after ICU admission. Associations with the primary and secondary outcomes were evaluated using univariable and multivariable logistic regression, and discriminative performance was assessed using receiver operating characteristic (ROC) curve analysis. Results: The in-hospital mortality rate was 56.2%. Log2-EASIX was independently associated with in-hospital mortality (OR: 1.191, 95% CI: 1.119–1.268; *p* < 0.001). ROC analysis showed moderate discrimination for predicting in-hospital mortality (AUC: 0.673, 95% CI: 0.648–0.698), with a ROC-derived exploratory cut-off of 1.47. Patients with log2-EASIX ≥ 1.47 had approximately a two-fold higher risk of in-hospital mortality (OR: 2.039, 95% CI: 1.610–2.584; *p* < 0.001). In multivariable analyses of the secondary outcomes, log2-EASIX remained independently associated with second-line vasopressor requirement (adjusted OR: 1.104, 95% CI: 1.040–1.171; *p* = 0.001) and CRRT requirement within 7 days (adjusted OR: 1.317, 95% CI: 1.236–1.404; *p* < 0.001). However, its associations with IMV and initial vasopressor requirements were not statistically significant after adjustment. Adding log2-EASIX to the Acute Physiology and Chronic Health Evaluation (APACHE) II resulted in a statistically significant but small improvement in discrimination compared with APACHE II alone (AUC: 0.761 vs. 0.740; ΔAUC = 0.021; 95% CI: 0.010–0.032; *p* < 0.001). Conclusions: EASIX was independently associated with in-hospital mortality and second-line vasopressor requirement, and it remained associated with CRRT requirement after multivariable adjustment. However, serum creatinine alone showed superior discrimination for CRRT, suggesting that the association between EASIX and CRRT may be driven, at least in part, by its creatinine component and should therefore be interpreted cautiously. In contrast, the associations of EASIX with early IMV and initial vasopressor requirements were not significant after multivariable adjustment. Although its discriminative performance for mortality was moderate when used alone, EASIX may provide complementary prognostic information when added to established disease severity assessment. These findings warrant validation in prospective, multicenter studies.

## 1. Introduction

Sepsis is a complex clinical syndrome marked by life-threatening organ dysfunction caused by a dysregulated host response to infection. It remains a leading cause of morbidity and mortality among critically ill patients admitted to intensive care units (ICUs) worldwide [1,2,3]. Pneumonia is the most common primary source of infection in patients with sepsis and is the leading cause of pneumosepsis, one of the most prevalent sepsis subtypes encountered in intensive care practice [4]. The systemic inflammatory response triggered by pulmonary infection may lead to severe clinical conditions, including acute hypoxemic respiratory failure, septic shock, and multiple organ dysfunction [5]. Therefore, in patients with pneumosepsis, early identification of both the risk of mortality and the need for organ support therapies, including invasive mechanical ventilation (IMV), vasopressor therapy, and continuous renal replacement therapy (CRRT), is crucial for optimizing patient management and the efficient allocation of ICU resources [2]. Although currently available prognostic scoring systems are useful for assessing disease severity, they do not directly reflect endothelial dysfunction, a key pathophysiological mechanism underlying sepsis [6,7].

Endothelial dysfunction is a key component of sepsis pathophysiology and central to the development of microcirculatory disturbances, increased vascular permeability, coagulation activation, and organ hypoperfusion. The Endothelial Activation and Stress Index (EASIX), calculated from serum lactate dehydrogenase (LDH), serum creatinine, and platelet count (PLT), is a simple, readily applicable biomarker that indirectly reflects multiple aspects of endothelial injury. Because it is derived from routinely available laboratory parameters, EASIX can be easily implemented in clinical practice. Previous studies have demonstrated its prognostic value across a wide range of clinical settings, including hematologic malignancies, coronavirus disease-2019 (COVID-19), and other critical illnesses. More recently, accumulating evidence shows that EASIX is independently associated with both short- and long-term mortality in patients with sepsis and may provide incremental prognostic information beyond conventional risk assessment tools [8,9,10,11].

However, prior evidence has demonstrated an association between EASIX and mortality in patients with pulmonary sepsis. Therefore, the association between EASIX and mortality in pneumosepsis should be considered confirmatory rather than entirely novel. Nevertheless, evidence regarding the relationship between EASIX and organ support requirements in this population remains limited. In particular, its associations with the need for IMV, vasopressor therapy, second-line vasopressor therapy, and CRRT have not been comprehensively evaluated. Furthermore, the incremental discriminative value of EASIX when added to an established disease severity score such as Acute Physiology and Chronic Health Evaluation (APACHE) II remains unclear. Accordingly, the present study aimed to confirm the previously reported association between EASIX and mortality in pneumosepsis and to extend existing evidence by evaluating its associations with organ support requirements and its incremental prognostic performance beyond APACHE II.

## 2. Materials and Methods

### 2.1. Study Design and Study Population

This retrospective observational cohort study used electronic medical records of patients admitted to the tertiary ICU of Sultan 2. Abdulhamid Han Training and Research Hospital, University of Health Sciences, from 1 January 2018 to 30 December 2025. Adult patients (≥18 years) admitted to the ICU with pneumonia who met the Sepsis-3 criteria for sepsis were eligible for inclusion.

**Figure 1:**
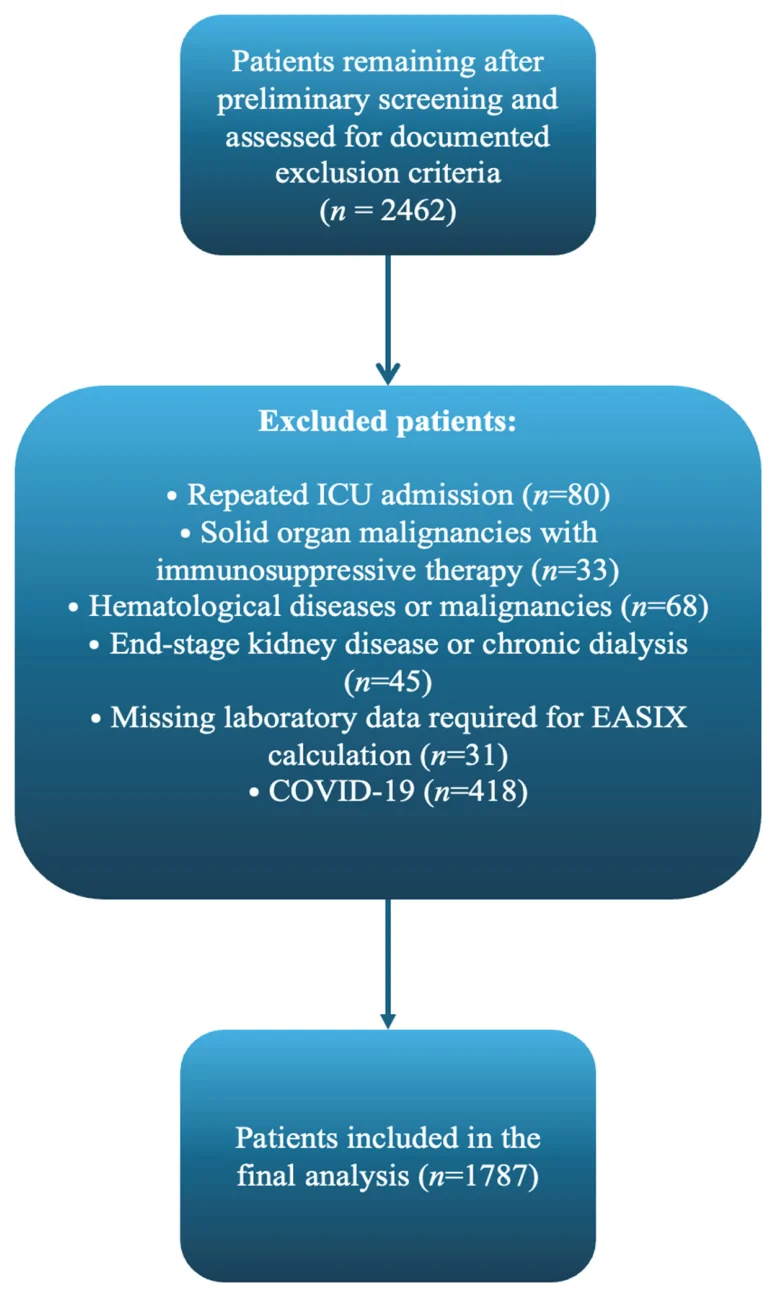
Study Flowchart. Patients with an ICU stay <24 h and those with a primary infection other than pneumonia were excluded during preliminary screening; the numbers excluded at this stage were not systematically recorded and could not be reliably reconstructed retrospectively. ICU: Intensive Care Unit, COVID-19: Coronavirus Disease 2019.

For patients with multiple ICU admissions, only the first admission was included in the analysis. Patients were excluded if they had an ICU stay of less than 24 h; missing laboratory data required for EASIX calculation (LDH, serum creatinine, or PLT); a primary infection other than pneumonia; active hematologic malignancy; active solid-organ malignancy receiving chemotherapy or immunosuppressive therapy; primary hematologic disorders or other conditions that could substantially affect PLT; end-stage chronic kidney disease or a history of chronic dialysis; or acute hemolysis, rhabdomyolysis, acute myocardial infarction, severe hepatic failure, or disseminated metastatic disease. Additionally, patients with COVID-19 were excluded from the study. Some prespecified eligibility criteria, including an ICU stay of <24 h and a primary infection other than pneumonia, were applied during preliminary screening before the identification of the 2462 potentially eligible patients. Individual counts of patients excluded at this preliminary stage were not systematically recorded and therefore could not be reliably reconstructed retrospectively. Among the 2462 potentially eligible patients subsequently evaluated, 675 were excluded for the documented reasons shown in Figure 1, leaving 1787 patients in the final analytical cohort.

Patients with an ICU stay of less than 24 h were excluded to ensure a sufficiently complete observation period for assessing early clinical outcomes and baseline disease severity. In addition, the complete admission data required for EASIX calculation and the prespecified analyses were not systematically available for these patients in the retrospective dataset.

### 2.2. Data Collection

Demographic characteristics (age and sex), presence of at least one comorbidity, vital signs at ICU admission (heart rate [HR] and peripheral oxygen saturation [SpO_2_]), laboratory parameters, and disease severity scores were retrospectively extracted from the electronic medical record system. Laboratory evaluation included complete blood count (white blood cell [WBC], hemoglobin [Hgb], and PLT); serum biochemistry (urea, serum creatinine, total bilirubin, albumin, pro-B-type natriuretic peptide [pro-BNP], LDH, and D-dimer); arterial blood gas analysis (pH, partial oxygen pressure [PaO_2_], partial carbon dioxide pressure [PaCO_2_], base excess [BE], bicarbonate [HCO_3_], and lactate); coagulation parameters (international normalized ratio [INR] and fibrinogen); and inflammatory biomarkers (C-reactive protein [CRP] and procalcitonin [PCT]). ICU LOS was also recorded.

### 2.3. Definitions

EASIX was computed using the following formula:

EASIX = [LDH (U/L) × serum creatinine (mg/dL)]/PLT (10^9^/L) [12].

Because the EASIX distribution was right-skewed, a logarithmic transformation was applied, and log2-EASIX was used in the regression analyses. In addition, based on the receiver operating characteristics (ROC)-derived cut-off identified using the Youden index, patients were categorized as low log2-EASIX (<1.47) or high log2-EASIX (≥1.47).

Sepsis was defined according to the Sepsis-3 criteria as suspected or confirmed infection accompanied by an increase of at least 2 points in the Sequential Organ Failure Assessment (SOFA) score at ICU admission. Pneumonia was diagnosed based on compatible clinical findings and radiographic evidence of a pulmonary infiltrate consistent with pneumonia. Pneumosepsis was defined as sepsis that met the Sepsis-3 criteria, with pneumonia identified as the primary source of infection.

Disease severity was assessed using the APACHE II and SOFA scores, calculated within the first 24 h after ICU admission.

The need for IMV was defined as endotracheal intubation or initiation of IMV within the first 24 h after ICU admission.

Vasopressor therapy was required if norepinephrine was initiated within the first 24 h after ICU admission to maintain a mean arterial pressure (MAP) of ≥65 mmHg. Second-line vasopressor therapy was required if an additional vasopressor was initiated because the target MAP could not be achieved despite a norepinephrine dose exceeding 0.25 μg/kg/min.

Second-line vasopressor therapy was defined as the addition of epinephrine to ongoing vasopressor treatment. Epinephrine was the second-line vasopressor used in all patients who required an additional vasopressor, as vasopressin was not routinely available in our clinical setting during the study period. Second-line vasopressor therapy was assessed throughout the entire ICU stay rather than being restricted to the first 24 h after ICU admission.

CRRT requirement was defined as initiation of CRRT within the first 7 days after ICU admission. CRRT was initiated at the treating ICU team’s discretion for conventional clinical indications, including refractory hyperkalemia, severe metabolic acidosis refractory to medical therapy, refractory fluid overload, persistent oliguria or anuria with progressive renal dysfunction, and/or clinically significant uremic complications.

### 2.4. Study Outcomes

The primary outcome was all-cause in-hospital mortality. Secondary outcomes included the need for IMV within the first 24 h after ICU admission, the need for vasopressor therapy within the first 24 h and second-line vasopressor therapy, and the need for CRRT within the first 7 days after ICU admission.

### 2.5. Statistical Analysis

Statistical analyses were conducted using IBM SPSS Statistics version 30.0 (IBM Corp., Armonk, NY, USA). The distribution of continuous variables was assessed using the Kolmogorov–Smirnov test and visual inspection of histograms. Normally distributed variables are presented as mean ± standard deviation (SD), whereas non-normally distributed variables are presented as median (interquartile range [IQR]). Categorical variables are expressed as frequencies and percentages.

Comparisons of continuous variables between two groups were performed using the independent-samples *t* test or the Mann–Whitney U test, as appropriate. Categorical variables were compared using the chi-square test or Fisher’s exact test.

Missing data were not imputed. Patients with missing LDH, serum creatinine, or PLT count values required for EASIX calculation were excluded from the study; therefore, EASIX was available for all patients in the final cohort. For other variables, analyses were performed using the available complete data for the variables included in each analysis.

Variables associated with in-hospital mortality were initially evaluated using univariable logistic regression. Multivariable models were then constructed using a limited set of clinically relevant covariates selected for their established prognostic relevance in sepsis and the biological domains represented by the variables, rather than solely on statistical significance in univariable analyses. Model 1 included age, sex, the presence of at least one comorbidity, serum albumin, serum lactate, and log2-EASIX to assess the association between log2-EASIX and mortality after adjustment for major demographic, comorbidity-related, nutritional, and metabolic severity-related factors.

Model 2 also included APACHE II to assess whether the association between log2-EASIX and mortality persisted after adjusting for overall disease severity. Because APACHE II incorporates age, chronic health information, and several physiological variables, there was partial conceptual overlap with some covariates in Model 1. Accordingly, Model 2 was interpreted as a severity-adjusted association model rather than a parsimonious prediction model. Model 3 had the same structure as Model 2, except that continuous log2-EASIX was replaced with the ROC-derived dichotomized EASIX variable (high vs. low) to evaluate the association of the clinically interpretable EASIX threshold with mortality.

For the secondary outcomes, univariable logistic regression analyses were first performed for IMV requirement within 24 h, vasopressor requirement within 24 h, second-line vasopressor requirement, and CRRT requirement within 7 days. Separate multivariable logistic regression models were then constructed for each secondary outcome using clinically relevant covariates that reflect baseline disease severity and outcome-specific physiological or organ-related disturbances. The IMV model included sex, pH, PaCO_2_, lactate, APACHE II, and log2-EASIX. The vasopressor and second-line vasopressor models included pH, lactate, APACHE II, and log2-EASIX. The CRRT model included the presence of at least one comorbidity, serum urea, APACHE II, and log2-EASIX. Variables representing overlapping physiological domains were not entered simultaneously, and individual components of EASIX (creatinine, LDH, and PLT) were excluded from the multivariable models to avoid redundant adjustment with the index itself.

Multicollinearity was assessed using the variance inflation factor (VIF) and tolerance. Model performance was evaluated using the Omnibus test, Nagelkerke R^2^, and the Hosmer–Lemeshow goodness-of-fit test.

The discriminative performance of EASIX for in-hospital mortality, IMV, vasopressor therapy, second-line vasopressor therapy, and CRRT was evaluated using ROC curve analysis. The area under curve (AUC) and corresponding 95% confidence intervals (CIs) were reported. For statistically significant ROC analyses, ROC-derived cut-off values were determined using the Youden index, and the corresponding sensitivity and specificity were reported. To assess the incremental discriminative value of log2-EASIX beyond APACHE II, the AUC of the APACHE II model was compared with that of the combined log2-EASIX + APACHE II model using DeLong’s test for correlated ROC curves. In addition, because serum creatinine is a component of EASIX and is directly related to renal dysfunction, the discriminative performance of serum creatinine alone for CRRT requirement was compared with that of log2-EASIX using DeLong’s test for correlated ROC curves.

Internal validation of the APACHE II and combined log2-EASIX + APACHE II mortality models was performed using bootstrap resampling with 2000 repetitions. For each bootstrap sample, the model was refitted, and its performance was evaluated in both the bootstrap sample and the original dataset. Optimism was estimated as the mean difference between bootstrap-sample and original-sample performance, and optimism-corrected estimates were obtained by subtracting the estimated optimism from the apparent performance. This procedure was applied to the AUC as well as to the calibration intercept and slope. Internal validation was restricted to patients with complete data on in-hospital mortality, APACHE II, and log2-EASIX. The calibration intercept and slope from the original development cohort were considered apparent calibration estimates, whereas optimism-corrected calibration estimates were derived from the bootstrap procedure. An ideal calibration intercept and slope were considered to be 0 and 1, respectively.

Decision-curve analysis was performed to assess the potential clinical utility of adding log2-EASIX to APACHE II by comparing the net benefit of APACHE II alone with that of the combined log2-EASIX + APACHE II model across threshold probabilities ranging from 0.10 to 0.80.

Categorical predictor variables were entered as binary indicator variables. Female sex was used as the reference category for sex, absence of comorbidity as the reference category for comorbidity status, and low log2-EASIX (<1.47) as the reference category for the dichotomized EASIX variable. Binary clinical outcomes were coded 0 = absence and 1 = presence, with the presence of each outcome modeled as the event.

All statistical tests were two-tailed, and a *p*-value <0.05 was considered statistically significant.

## 3. Results

### 3.1. Baseline Characteristics of the Study Population

In the present study, the term “pneumosepsis” refers to sepsis in which pneumonia is identified as the primary source of infection. A total of 1787 patients with pneumosepsis admitted to the ICU were included in the study. The median age was 69 years (57–78), and 998 (55.8%) were male. The overall in-hospital mortality rate was 56.2% (*n* = 1004). Within the first 24 h after ICU admission, 88.2% of patients required IMV and 93.0% received vasopressor therapy. During the ICU stay 30.8% required second-line vasopressor therapy. CRRT was initiated within the first 7 days in 31.6% of patients. The median ICU length of stay (LOS) was 5.88 days (2.04–12.71).

The median APACHE II and SOFA scores were 28 (22–34) and 10 (7–13), respectively. The median EASIX score was 2.46 (1.02–6.70), and the median log2-EASIX score was 1.29 (0.02–2.74). At ICU admission, the median lactate level was 2.20 mmol/L (1.40–4.29), serum creatinine was 1.47 mg/dL (0.84–2.55), PLT was 227 × 10^9^/L (140–315), and LDH level was 317 U/L (230–508). Detailed demographic, clinical, and laboratory characteristics of the study population are presented in Table 1.

### 3.2. Comparison of Patients According to In-Hospital Mortality

Compared with survivors, patients who died during hospitalization were significantly older, and sex distribution differed significantly between the two groups (both *p* < 0.05). The presence of at least one comorbidity was more frequent among non-survivors than among survivors (22.4% vs. 18.0%, *p* = 0.022). In addition, non-survivors had a significantly shorter ICU LOS (*p* < 0.001) (Table 2).

Patients who died during hospitalization were more likely than survivors to require vasopressor therapy within the first 24 h, second-line vasopressor therapy and CRRT within the first 7 days (all *p* < 0.001) (Table 2). In contrast, the requirement for IMV within the first 24 h was significantly more frequent among survivors (*p* = 0.033).

With respect to laboratory findings, non-survivors had significantly higher lactate, urea, creatinine, CRP, PCT, LDH, INR, and D-dimer levels, whereas albumin, Hgb, PLT, and BE were significantly lower than those of survivors (all *p* < 0.05) (Table 2, Supplementary Table S1).

Disease severity scores, including APACHE II and SOFA, as well as the endothelial injury markers EASIX and log2-EASIX, were significantly higher in non-survivors than in survivors (all *p* < 0.001). Furthermore, the proportion of patients with a high log2-EASIX score (≥1.47) was significantly greater among non-survivors (*p* < 0.001) (Table 2).

### 3.3. Discriminative Performance of log2-EASIX for In-Hospital Mortality

ROC curve analysis showed that log2-EASIX had a statistically significant but moderate ability to predict in-hospital mortality (AUC: 0.673, 95% CI: 0.648–0.698; *p* < 0.001). The ROC-derived cut-off identified by the Youden index was 1.47 (corresponding to a raw EASIX value of 2.77), yielding a sensitivity of 59% (95% CI: 56–62%), a specificity of 70% (95% CI: 67–73%), a positive predictive value (PPV) of 71% (95% CI: 68–74%), and a negative predictive value (NPV) of 57% (95% CI: 54–60%) (Table 3). Using this cut-off, patients were categorized into high log2-EASIX (≥1.47) and low log2-EASIX (<1.47) groups. This classification was used in all subsequent comparative and regression analyses.

### 3.4. Comparison According to log2-EASIX Groups

Using the ROC-derived cut-off value, patients were categorized into high log2-EASIX (≥1.47, *n* = 824) and low log2-EASIX (<1.47, *n* = 963) groups. The in-hospital mortality rate was significantly higher in the high log2-EASIX group than in the low log2-EASIX group (71.4% vs. 43.2%, *p* < 0.001). In addition, patients with high log2-EASIX were more likely to require vasopressor therapy within the first 24 h (96.0% vs. 90.4%), second-line vasopressor therapy (40.7% vs. 22.4%), and CRRT within the first 7 days (48.1% vs. 17.4%) (all *p* < 0.001). In contrast, the two groups did not differ significantly in the need for IMV within the first 24 h (*p* = 0.355) (Table 4).

The proportion of patients with at least one comorbidity was significantly higher in the high log2-EASIX group than in the low log2-EASIX group (27.3% vs. 14.6%, *p* < 0.001). Laboratory analysis showed that patients with high log2-EASIX had significantly higher lactate, urea, creatinine, total bilirubin, CRP, PCT, LDH, D-dimer, and INR levels, whereas Hgb, PLT, and HCO_3_ levels were significantly lower (all *p* < 0.001). In contrast, age, sex, PaO_2_, WBC, and HR did not differ significantly between the two groups (*p* > 0.05) (Table 4, Supplementary Table S2).

Disease severity scores, including APACHE II and SOFA, were significantly higher in the high log2-EASIX group than in the low log2-EASIX group (both *p* < 0.001) (Table 4).

### 3.5. Factors Associated with In-Hospital Mortality

In the univariate logistic regression analysis, age, sex, the presence of at least one comorbidity, IMV requirement within the first 24 h, vasopressor therapy within the first 24 h, second-line vasopressor therapy, CRRT within the first 7 days, ICU LOS, pH, BE, HCO_3_, PaCO_2_, lactate, urea, creatinine, total bilirubin, PCT, Hgb, PLT, albumin, pro-BNP, LDH, D-dimer, INR, fibrinogen, SpO_2_, HR, APACHE II, SOFA, and log2-EASIX score were all significantly associated with in-hospital mortality (all *p* < 0.05). In contrast, CRP was not significantly associated with in-hospital mortality (*p* = 0.137). The dichotomized high log2-EASIX variable (≥1.47), defined by the ROC-identified cut-off, was also strongly associated with in-hospital mortality (OR: 3.276, 95% CI: 2.689–3.992; *p* < 0.001) (Table 5).

In the multivariable logistic regression analysis, all three models were statistically significant by the Omnibus test (all *p* < 0.001). In Model 1, log2-EASIX was independently associated with in-hospital mortality (OR: 1.275, 95% CI: 1.203–1.350; *p* < 0.001). In Model 2, the association between log2-EASIX and in-hospital mortality remained statistically significant after adjusting for APACHE II (OR: 1.191, 95% CI: 1.119–1.268; *p* < 0.001). In Model 3, in which the continuous log2-EASIX variable was replaced with the dichotomized high log2-EASIX variable (≥1.47), derived from the ROC-defined cut-off value, high log2-EASIX remained independently associated with in-hospital mortality (OR: 2.039, 95% CI: 1.610–2.584; *p* < 0.001) (Table 6).

Regarding model performance, adding APACHE II markedly improved the models’ explanatory power. The Nagelkerke R^2^ values were 0.221, 0.320, and 0.323 for Models 1, 2, and 3, respectively. The Hosmer–Lemeshow goodness-of-fit test indicated good calibration for Model 2 (*p* = 0.065) and Model 3 (*p* = 0.095). Furthermore, multicollinearity diagnostics showed VIF values below 2.0 and tolerance values above 0.80 for all variables included in the models (Table 6).

### 3.6. Incremental Prognostic Value of log2-EASIX Beyond APACHE II

**Figure 2:**
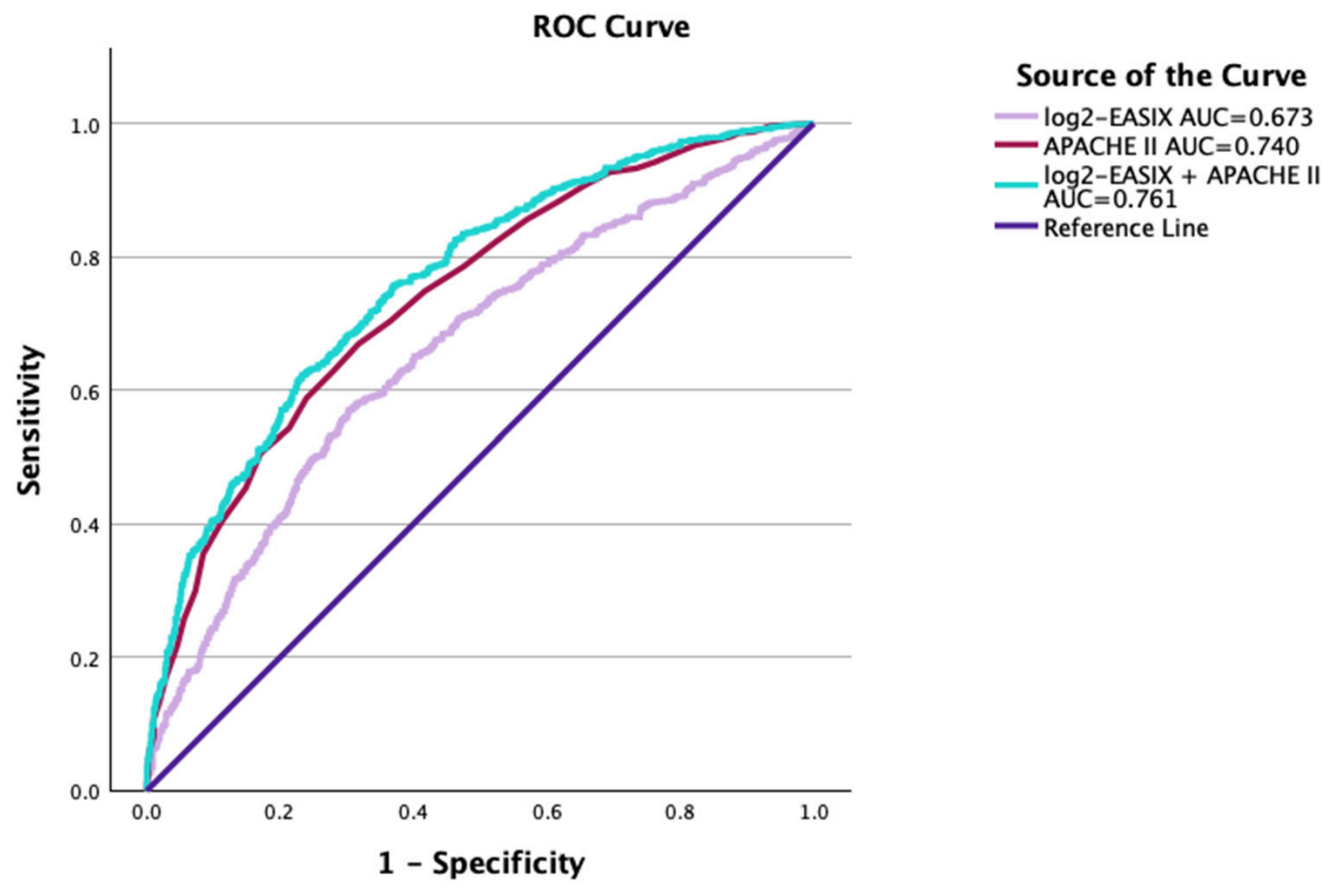
ROC curves comparing the discriminative performance of log2-EASIX, APACHE II, and the combined model (log2-EASIX + APACHE II) for in-hospital mortality. The combined model demonstrated the highest discriminative ability (AUC = 0.761), followed by APACHE II (AUC = 0.740) and log2-EASIX alone (AUC = 0.673). According to the DeLong test, the combined model significantly outperformed the APACHE II model alone (ΔAUC = 0.021, 95% CI: 0.010–0.032, *p* < 0.001).

ROC curve analysis showed that log2-EASIX demonstrated moderate discriminative performance for in-hospital mortality (AUC: 0.673, 95% CI: 0.648–0.698). APACHE II demonstrated better discriminative performance, with an AUC of 0.740 (95% CI: 0.716–0.764). The combined model incorporating both log2-EASIX and APACHE II achieved the highest discriminative performance, with an AUC of 0.761 (95% CI: 0.738–0.784) (Table 7, Figure 2).

Regarding model performance, the combined model demonstrated greater explanatory power than the models using log2-EASIX or APACHE II alone. The Nagelkerke R^2^ values were 0.118 for the log2-EASIX model, 0.230 for the APACHE II model, and 0.267 for the combined model. The Omnibus test was statistically significant for all models (*p* < 0.001), and the Hosmer–Lemeshow goodness-of-fit test indicated acceptable model calibration (Table 7).

DeLong’s test showed a statistically significant but modest improvement in discrimination with the combined log2-EASIX + APACHE II model compared with APACHE II alone (ΔAUC = 0.021, 95% CI: 0.010–0.032; *p* < 0.001) (Table 7, Figure 2).

**Figure 3:**
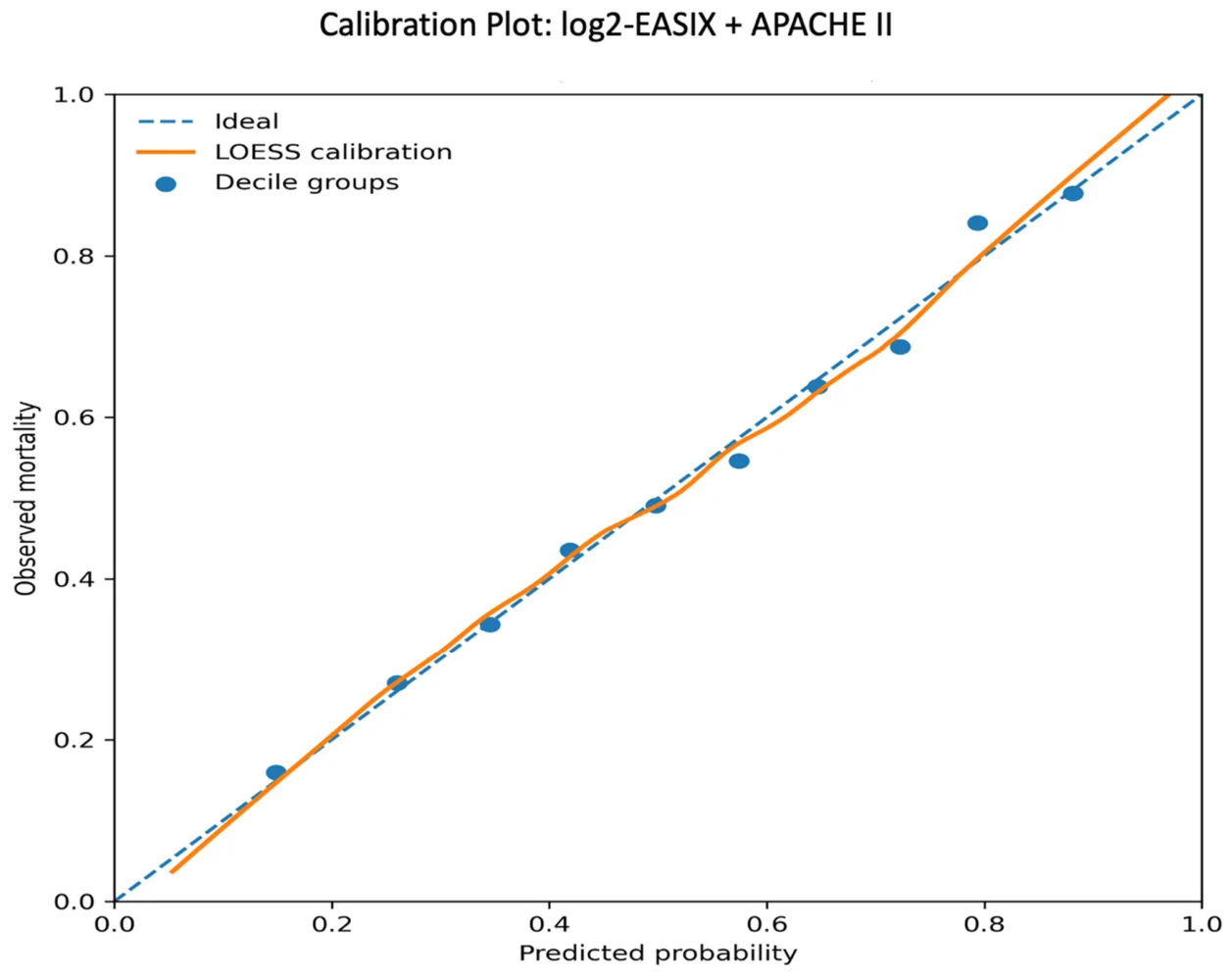
Apparent calibration plot for the combined log2-EASIX and APACHE II model predicting in-hospital mortality. The diagonal line represents perfect calibration. The calibration curve shows agreement between predicted and observed probabilities of in-hospital mortality.

Internal validation was performed in 1630 patients with complete data on in-hospital mortality, APACHE II, and log2-EASIX. After 2000 bootstrap resamples, optimism was minimal for both the APACHE II model (optimism = 0.00012) and the combined log2-EASIX + APACHE II model (optimism = 0.00049). The corresponding optimism-corrected AUCs were 0.740 and 0.761, respectively, yielding an optimism-corrected AUC difference of 0.021. The apparent calibration intercept and slope of the combined model were approximately 0 and 1, respectively (Figure 3). After internal validation with 2000 bootstrap resamples, the optimism-corrected calibration intercept and slope were 0.001 and 0.999, respectively, indicating minimal calibration optimism.

**Figure 4:**
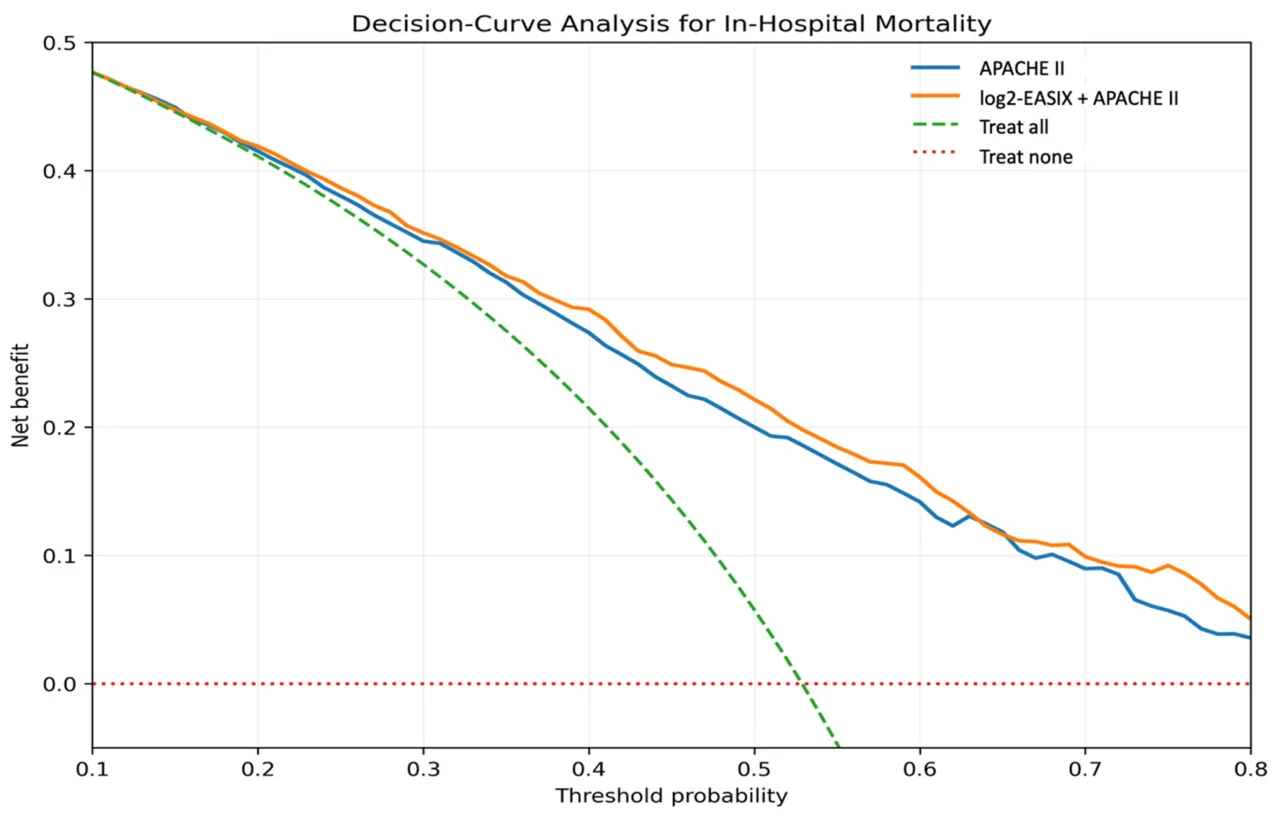
Decision-curve analysis comparing APACHE II and the combined log2-EASIX + APACHE II model for predicting in-hospital mortality. Across most threshold probabilities from 0.10 to 0.80, the combined model yielded a modestly higher net benefit than APACHE II alone. The “treat all” and “treat none” strategies are shown for reference.

Decision-curve analysis showed that the combined log2-EASIX + APACHE II model provided a modestly higher net benefit than APACHE II alone across most evaluated threshold probabilities, although the incremental clinical benefit was limited (Figure 4).

### 3.7. Association of log2-EASIX with Secondary Clinical Outcomes

In univariable logistic regression analyses, log2-EASIX was significantly associated with IMV requirement within the first 24 h (OR: 1.074, 95% CI: 1.001–1.152; *p* = 0.047), vasopressor requirement within the first 24 h (OR: 1.233, 95% CI: 1.117–1.360; *p* < 0.001), second-line vasopressor requirement (OR: 1.262, 95% CI: 1.202–1.325; *p* < 0.001), and CRRT requirement within the first 7 days (OR: 1.365, 95% CI: 1.297–1.437; *p* < 0.001) (Table 8).

After adjustment for outcome-specific clinically relevant covariates, log2-EASIX remained independently associated with second-line vasopressor requirement (adjusted OR: 1.104, 95% CI: 1.040–1.171; *p* = 0.001) and CRRT requirement within the first 7 days (adjusted OR: 1.317, 95% CI: 1.236–1.404; *p* < 0.001). In contrast, the associations of log2-EASIX with IMV requirement (adjusted OR: 0.961, 95% CI: 0.888–1.041; *p* = 0.334) and vasopressor requirement (adjusted OR: 1.036, 95% CI: 0.925–1.161; *p* = 0.541) were no longer statistically significant after multivariable adjustment (Table 8).

The discriminative performance of log2-EASIX for the secondary outcomes was evaluated using ROC curve analysis. The discriminative ability of log2-EASIX for predicting the need for IMV within the first 24 h was not statistically significant (AUC: 0.539, 95% CI: 0.497–0.581; *p* = 0.066). In contrast, log2-EASIX showed statistically significant discriminative performance for vasopressor requirement within the first 24 h (AUC: 0.618, 95% CI: 0.570–0.666; *p* < 0.001), second-line vasopressor need (AUC: 0.641, 95% CI: 0.614–0.669; *p* < 0.001), and CRRT requirement within the first 7 days (AUC: 0.712, 95% CI: 0.687–0.736; *p* < 0.001). The ROC-derived cut-off values determined by the Youden index were 1.46 for vasopressor therapy, 1.89 for second-line vasopressor therapy, and 1.41 for CRRT requirement (Table 9).

In a secondary analysis assessing the potential influence of the creatinine component of EASIX, serum creatinine alone demonstrated greater discriminative ability for CRRT requirement than log2-EASIX (AUC: 0.790, 95% CI: 0.767–0.813 vs. 0.712, 95% CI: 0.687–0.736). DeLong’s test confirmed that this difference was statistically significant (ΔAUC = 0.078, 95% CI: 0.057–0.100; *p* < 0.001).

## 4. Discussion

In this retrospective cohort study, we confirmed the previously reported association between higher EASIX values and mortality in patients with pneumosepsis. Beyond this confirmatory finding, the present study extended the evaluation of EASIX to organ support requirements and its incremental discriminative value beyond APACHE II. After multivariable adjustment, log2-EASIX remained independently associated with the need for second-line vasopressor therapy and CRRT, whereas its associations with early IMV and initial vasopressor requirement were no longer statistically significant. In addition, combining log2-EASIX with APACHE II resulted in a statistically significant increase in mortality discrimination compared with APACHE II alone, although the magnitude and clinical relevance of this incremental improvement require cautious interpretation.

A fundamental pathophysiological feature of sepsis is widespread endothelial activation and dysfunction driven by the dysregulated host response to infection. Under physiological conditions, the endothelium is a dynamic organ that plays a central role in regulating vascular tone, maintaining microvascular barrier integrity, modulating the inflammatory response, and preserving hemostatic balance. During the early phase of sepsis, endothelial activation is considered an adaptive response that facilitates the recruitment and migration of immune cells to the site of infection. However, as the inflammatory response becomes excessive and dysregulated, endothelial activation shifts from a protective to a maladaptive process [13]. Progressive degradation of the endothelial glycocalyx, increased vascular permeability, platelet activation, microthrombosis, and impaired microcirculatory perfusion ultimately result in tissue hypoperfusion and the development of multiple organ dysfunction [8]. Accordingly, sepsis is now widely recognized not merely as a consequence of infection or systemic inflammation but as a syndrome marked by profound endothelial dysfunction. As the extent of endothelial injury increases, so does the severity of organ dysfunction and the risk of mortality. Therefore, early assessment of endothelial activation may play an important role in risk stratification and in identifying patients who may benefit from future endothelium-targeted therapeutic strategies [14]. The growing interest in therapeutic strategies to preserve endothelial barrier integrity has further underscored the need for biomarkers that can assess endothelial activation at an early stage [8,15].

Despite the well-established central role of endothelial dysfunction in sepsis prognosis, the routine clinical use of biomarkers that directly assess endothelial injury remains limited [7]. Although angiopoietin-2 (Ang-2), soluble thrombomodulin, syndecan-1, endocan, and other inflammatory mediators have been identified as promising biomarkers of endothelial injury, their widespread adoption in routine clinical practice has been limited by high costs, the need for specialized laboratory infrastructure, and the lack of standardized analytical methods [6,7,10]. In contrast, the EASIX score is derived entirely from routinely measured laboratory parameters, namely serum LDH, serum creatinine, and PLT, making it readily calculable without additional costs or specialized laboratory assays [16]. Furthermore, EASIX has been reported to correlate significantly with biomarkers of endothelial activation, including Ang-2, soluble thrombomodulin, ST2, CXCL8, and insulin-like growth factor-1 (IGF-1). These findings support the notion that EASIX may serve as an indirect yet reliable surrogate marker of endothelial injury [16,17]. Given its simplicity and low cost, EASIX may be a valuable tool for early risk stratification, particularly in resource-limited healthcare settings. Its ease of calculation from routinely available laboratory parameters may be especially advantageous in clinical situations where rapid risk assessment at ICU admission is essential.

In the present study, EASIX was independently associated with in-hospital mortality, consistent with previous reports. Xu et al. demonstrated, in a retrospective analysis of the MIMIC-IV database, that higher log2-EASIX values were independently associated with 28- and 90-day mortality among patients with sepsis [9]. Similarly, Li et al. demonstrated in a larger cohort of patients with sepsis that EASIX showed a linear association not only with short-term mortality but also with 30-, 60-, 90-, 180-, and 1-year mortality [10]. Likewise, in a two-cohort study of patients with pulmonary sepsis, Yang et al. demonstrated that EASIX independently predicted 28-day mortality and that higher EASIX levels were associated with higher APACHE II and SOFA scores [11]. Accordingly, our finding that higher EASIX values are associated with increased in-hospital mortality should primarily be interpreted as confirmation of prior observations in pulmonary sepsis rather than as an entirely novel association. The present study’s additional contribution is extending the evaluation of EASIX beyond mortality to organ support requirements and formally assessing its incremental discriminative performance beyond APACHE II. Importantly, after adjusting for baseline disease severity and outcome-specific covariates, log2-EASIX remained independently associated with second-line vasopressor and CRRT requirements but not with initial vasopressor or IMV requirements. Furthermore, adding log2-EASIX to APACHE II produced a statistically significant increase in AUC; however, the absolute increase was small, and its clinical relevance remains uncertain. These findings therefore support EASIX as a potentially complementary prognostic marker rather than a replacement for established disease severity scores. While APACHE II primarily reflects physiological derangement and overall disease severity, EASIX may be more closely related to endothelial activation and dysfunction. Therefore, the complementary pathophysiological information captured by these two indices may explain their improved prognostic performance when used together.

Each of the three components of the EASIX score reflects distinct yet interrelated aspects of sepsis pathophysiology. LDH is widely recognized as a marker of cellular injury and tissue hypoxia, whereas serum creatinine reflects renal hypoperfusion and impaired kidney function, both of which are among the earliest signs of organ dysfunction in sepsis. A reduced PLT, in turn, is considered an important indicator of endothelial activation, thromboinflammation, and microvascular thrombosis [11,18]. By integrating these three parameters into a single score, EASIX enables simultaneous assessment of cellular injury, organ dysfunction, and coagulation abnormalities, which are key pathophysiological processes underlying sepsis [10]. This strong biological rationale may, at least in part, explain EASIX’s prognostic value in patients with sepsis.

In univariable analyses, higher log2-EASIX was associated with both initial and second-line vasopressor requirements. However, these associations differed after adjustment for baseline disease severity and hemodynamic variables. Log2-EASIX remained independently associated with second-line vasopressor requirement, whereas its association with initial vasopressor use within the first 24 h was no longer statistically significant. Circulatory failure in sepsis results from systemic vasodilation, increased vascular permeability, intravascular volume depletion, and impaired microcirculatory perfusion, processes in which endothelial activation and dysfunction play central roles [19,20]. Previous studies have also linked EASIX to hemodynamic instability and vasopressor requirements in critically ill patients [6]. Our adjusted findings therefore suggest that EASIX may be more closely associated with the severity or progression of hemodynamic dysfunction, as reflected by the need for second-line vasopressor support, than with the initial decision to start vasopressor therapy. Nevertheless, this finding should be interpreted cautiously and requires confirmation in prospective studies.

Another important finding was that log2-EASIX remained independently associated with the need for CRRT during the first 7 days after adjustment for comorbidity, serum urea, and baseline disease severity as assessed by APACHE II. Owing to its rich microvascular network, the kidney is among the organs most susceptible to hypoperfusion and endothelial injury during sepsis [8,21]. Sepsis-induced endothelial dysfunction contributes to the development of acute kidney injury by disrupting glomerular microcirculation, increasing capillary permeability, and reducing renal perfusion [22,23,24]. However, interpreting the association between EASIX and CRRT requires particular caution because serum creatinine, one of the three components of EASIX, is directly related to renal dysfunction and to the decision to initiate renal replacement therapy. In our additional ROC analysis, serum creatinine alone showed significantly better discrimination for CRRT requirement than log2-EASIX (AUC: 0.790 vs. 0.712; ΔAUC = 0.078; DeLong *p* < 0.001). This finding suggests that the observed association between EASIX and CRRT may be driven, at least in part, by its creatinine component rather than by additional information provided by the composite index or by endothelial dysfunction alone. Recent studies have shown that higher EASIX values are associated with adverse outcomes among critically ill patients receiving CRRT [25]. However, our findings do not demonstrate that EASIX is superior to conventional renal markers in identifying patients who will require CRRT. Therefore, the potential role of EASIX in renal risk assessment and CRRT-related clinical decision-making requires further prospective validation.

In univariable analysis, log2-EASIX showed a weak association with IMV requirement within the first 24 h; however, this association was no longer statistically significant after adjusting for sex, pH, PaCO_2_, lactate, and APACHE II. Consistent with this finding, its discriminative performance on ROC analysis was poor, and no significant difference in IMV requirement was observed between the high- and low-log2-EASIX groups. Taken together, these findings do not support an independent association between EASIX and early IMV need.

One possible explanation is that the decision to initiate IMV in patients with pneumosepsis is influenced not only by systemic endothelial injury but also by multiple respiratory and clinical factors, including the severity of hypoxemia, work of breathing, level of consciousness, airway secretion management, and clinical judgment. Accordingly, although EASIX appears to be more closely associated with circulatory and renal dysfunction, it may not provide sufficient discriminatory information for the IMV requirement when considered alone. The finding that IMV use within the first 24 h was slightly more frequent among survivors than among non-survivors should also be interpreted cautiously. Because patients with an ICU stay of less than 24 h were excluded, very early deaths may have been underrepresented in the analyzed cohort. This exclusion could have introduced survivorship or selection bias, particularly for outcomes defined within the first 24 h, and may have contributed to the unexpected distribution of IMV use between survivors and non-survivors. Therefore, this finding should not be interpreted as indicating a protective association between IMV and mortality.

Our findings suggest that EASIX should be considered a complementary prognostic biomarker rather than a replacement for established disease severity scores [6,25]. Because EASIX can be readily calculated from routinely available laboratory parameters at no additional cost, it may have practical advantages as an accessible prognostic marker, particularly in resource-limited intensive care settings [26]. In addition, EASIX may have potential relevance for identifying patients who warrant closer clinical monitoring and for future investigation of endothelium-targeted therapeutic strategies [24]. However, in the present study, the observed improvement in discrimination beyond APACHE II was modest (ΔAUC = 0.021). Consistent with this modest improvement in discrimination, decision-curve analysis showed only limited additional net benefit of the combined log2-EASIX + APACHE II model compared with APACHE II alone across the evaluated threshold probabilities. This statistically significant improvement should therefore not be interpreted as evidence of a substantial or established clinical benefit in risk stratification or patient management. Therefore, prospective external validation and studies specifically assessing clinical utility are required before EASIX can be recommended for routine clinical decision-making.

The ROC-derived log2-EASIX cut-off of 1.47 should also be interpreted cautiously. Because this threshold was identified and evaluated in the same cohort, the reported sensitivity and specificity may be optimistic. Accordingly, this cut-off should be considered exploratory rather than an established clinical threshold, and independent external validation is required before it can be used for clinical risk stratification.

## 5. Limitations

This study has several limitations. First, its retrospective, single-center design precludes causal inference and limits the generalizability of the findings. Second, EASIX was calculated solely from laboratory parameters obtained at ICU admission, and serial changes over time were not evaluated. In addition, treatment-related variables, such as fluid resuscitation, vasopressor doses, and the timing of antibiotic therapy, were not assessed; therefore, their potential impact on the study outcomes could not be analyzed. Furthermore, established biomarkers of endothelial activation or injury, including Ang-2, soluble thrombomodulin, ST2, CXCL8, and IGF-1, were not measured. Therefore, the present study cannot directly demonstrate that EASIX reflects endothelial injury or establish a biological correlation between EASIX and endothelial dysfunction. Accordingly, EASIX should be interpreted as a potential surrogate marker that may indirectly reflect endothelial injury rather than as a direct biomarker of endothelial damage. Finally, because our study included only patients with pneumosepsis, the findings should be validated in prospective, multicenter studies involving broader sepsis populations.

Another limitation, excluding patients with an ICU stay of less than 24 h, was intended to provide a sufficiently complete observation window for assessing organ support requirements and baseline clinical severity. However, this criterion may have introduced selection and survivorship bias by excluding patients who died very early or otherwise left the ICU within the first 24 h. Because detailed laboratory and outcome data for these excluded patients were not collected in the study dataset, their number and outcomes could not be reliably reconstructed, and a sensitivity analysis including them was not feasible. This limitation is particularly relevant to the interpretation of IMV and initial vasopressor requirements, both of which were defined within the first 24 h.

A further limitation concerns the interpretation of the association between EASIX and CRRT. Because serum creatinine is an integral component of EASIX and is directly related to renal dysfunction and the decision to initiate renal replacement therapy, the observed association between EASIX and CRRT may partly reflect the creatinine component of the index rather than additional information provided by the composite score. Consistent with this concern, serum creatinine alone demonstrated greater discriminative performance for CRRT requirement than log2-EASIX in the present cohort. In addition, detailed pre-ICU baseline renal function and serial renal data required for formal staging of acute kidney injury were not systematically collected in the study dataset; therefore, residual confounding by baseline renal function and acute kidney injury severity cannot be excluded. Accordingly, the association between EASIX and CRRT should not be interpreted as evidence of an endothelial-specific effect or as establishing EASIX as a tool for CRRT-related clinical decision-making.

In addition, EASIX is derived from laboratory values obtained at ICU admission, whereas APACHE II incorporates the worst physiological measurements recorded during the first 24 h. Therefore, the combined log2-EASIX + APACHE II model should not be interpreted as a purely admission-based prediction model, and the two predictors represent different time windows.

Several clinical characteristics were not systematically captured in the analytical dataset. Comorbidity information was available only as a binary variable indicating the presence or absence of at least one documented comorbidity; therefore, individual comorbid conditions and overall comorbidity burden could not be evaluated, and the relatively low prevalence of documented comorbidity should be interpreted cautiously. Pneumonia subtype (community-acquired, hospital-acquired, or ventilator-associated), microbiological confirmation and causative pathogens, source of ICU admission, and detailed treatment characteristics beyond the organ-support interventions evaluated in this study were also not systematically recorded and could not be reliably reconstructed retrospectively. Furthermore, the high rates of IMV, vasopressor requirement, and in-hospital mortality reflect a highly selected, severely ill population treated in a tertiary ICU, which may limit the generalizability of our findings to less severely ill patients and other ICU settings.

## 6. Conclusions

In conclusion, EASIX was independently associated with in-hospital mortality, second-line vasopressor use, and CRRT use in patients with pneumosepsis, whereas its associations with initial vasopressor and IMV use were not statistically significant after multivariable adjustment. Adding log2-EASIX to APACHE II resulted in a statistically significant but modest improvement in mortality discrimination (ΔAUC = 0.021), which remained stable after bootstrap internal validation. However, the clinical relevance of this small incremental improvement has not been established. Therefore, EASIX should currently be regarded as a complementary prognostic marker rather than a tool with demonstrated clinical utility, and prospective external validation is required before its routine use in clinical risk stratification can be recommended.

## Figures and Tables

**Table 1:** Baseline demographic, clinical, and laboratory characteristics of the study population.

Variables	Mean ± SD, Median (Q1–Q3) or *n* (%)
**Gender**	
Female	789 (44.2)
Male	998 (55.8)
Age, years	69.00 (57.00–78.00)
In-hospital mortality	1004 (56.2)
IMV need within 24 h	1576 (88.2)
CRRT need within 7 d	564 (31.6)
VP need within 24 h	1662 (93.0)
Second VP need	551 (30.8)
≥1 comorbidity	366 (20.5)
LOS ICU, days	5.88 (2.04–12.71)
pH	7.31 (7.21–7.41)
BE, mmol/L	−5.00 ± 8.15
HCO_3_, mmol/L	20.47 ± 6.34
PaO_2_, mmHg	88.45 (58.98–130.00)
PaCO_2_, mmHg	41.20 (33.70–51.00)
Lactate, mmol/L	2.20 (1.40–4.29)
Urea, mg/dL	75.30 (45.38–122.33)
Creatinine, mg/dL	1.47 (0.84–2.55)
Total bilirubin, mg/dL	0.66 (0.41–1.21)
CRP, mg/L	114.98 (35.00–218.00)
PCT, ng/mL	2.66 (0.68–14.65)
WBC × 10^9^/L	12.84 (7.97–18.85)
Hgb, g/dL	10.10 (8.60–11.80)
PLT × 10^9^/L	227.00 (140.00–315.00)
Albumin, g/L	27.55 ± 6.58
Pro-BNP, pg/mL	5964.00 (1656.00–20,127.00)
LDH, U/L	317.00 (230.00–508.00)
D-dimer, mg/L	3.38 (1.70–7.05)
INR	1.33 (1.16–1.62)
Fibrinogen, mg/dL	452.00 (323.00–592.00)
SpO_2_, %	94.00 (90.00–98.00)
HR, beats/min	106.00 (90.00–123.00)
APACHE II	28.00 (22.00–34.00)
SOFA	10.00 (7.00–13.00)
EASIX Score	2.46 (1.02–6.70)
log2-EASIX Score	1.29 (0.02–2.74)

IMV: Invasive Mechanical Ventilation, CRRT: Continuous Renal Replacement Therapy, VP: Vasopressor, LOS ICU: Length of Intensive Care Unit Stay, BE: Base Excess, PaO_2_: Partial Pressure of Oxygen, PaCO_2_: Partial Pressure of Carbon Dioxide, CRP: C-Reactive Protein, PCT: Procalcitonin, WBC: White Blood Cell, PLT: Platelet Count, Pro-BNP: Pro-B-type Natriuretic Peptide, LDH: Lactate Dehydrogenase, INR: International Normalized Ratio, SpO_2_: Peripheral Oxygen Saturation, HR: Heart Rate, APACHE II: Acute Physiology and Chronic Health Evaluation, SOFA: Sequential Organ Failure Assessment, EASIX: Endothelial Activation and Stress Index, log2-EASIX: Log2-transformed Endothelial Activation and Stress Index. The study cohort comprised 1787 patients. Missing data were present for the following baseline variables: age, *n* = 1 (0.1%); BE, *n* = 12 (0.7%); HCO_3_, *n* = 101 (5.7%); PaO_2_, *n* = 1 (0.1%); urea, *n* = 1 (0.1%); total bilirubin, *n* = 20 (1.1%); CRP, *n* = 47 (2.6%); PCT, *n* = 79 (4.4%); albumin, *n* = 17 (1.0%); pro-BNP, *n* = 1232 (68.9%); D-dimer, *n* = 748 (41.9%); INR, *n* = 39 (2.2%); fibrinogen, *n* = 676 (37.8%); SpO_2_, *n* = 320 (17.9%); HR, *n* = 327 (18.3%); APACHE II, *n* = 157 (8.8%); and SOFA, *n* = 301 (16.8%). All other variables presented in Table 1 had complete data. Categorical variables are presented as *n* (%), with percentages calculated using the total study population (*n* = 1787) as the denominator.

**Table 2:** Comparison of demographic characteristics, clinical outcomes, and severity-related parameters according to in-hospital mortality.

Variables	Non-Survivors Mean ± SD, Median (Q1–Q3) or *n* (%) *n* = 1004	Survivors Mean ± SD, Median (Q1–Q3) or *n* (%) *n* = 783	*p*-Value
**Gender**			0.014
Female	469 (46.7)	320 (40.9)	
Male	535 (53.3)	463 (59.1)	
Age, years	70.00 (58.00–77.00)	65.50 (52.00–77.00)	<0.001
IMV need within 24 h	871 (86.8)	705 (90.0)	0.033
CRRT need within 7 d	385 (38.3)	179 (22.9)	<0.001
VP need within 24 h	984 (98.0)	678 (86.6)	<0.001
Second VP need	504 (50.2)	47 (6.0)	<0.001
≥1 comorbidity	225 (22.4)	141 (18.0)	0.022
LOS ICU, days	3.56 (1.13–9.67)	8.25 (4.54–15.00)	<0.001
Lactate, mmol/L	2.20 (1.30–3.80)	1.80 (1.28–2.53)	<0.001
Albumin, g/L	26.49 ± 6.44	28.91 ± 6.44	<0.001
APACHE II	32.44 ± 7.01	25.21 ± 7.94	<0.001
SOFA	12.00 (10.00–14.00)	8.00 (5.00–11.00)	<0.001
EASIX Score	3.71 (1.22–9.05)	1.73 (0.77–3.72)	<0.001
log2-EASIX Score	1.89 (0.28–3.18)	0.79 (−0.39–1.89)	<0.001
log2-EASIX group			<0.001
High log2-EASIX (≥1.47)	588 (58.6)	236 (30.1)	
Low log2-EASIX (<1.47)	416 (41.4)	547 (69.9)	

IMV: Invasive Mechanical Ventilation, CRRT: Continuous Renal Replacement Therapy, VP: Vasopressor, LOS ICU: Length of Intensive Care Unit Stay, APACHE II: Acute Physiology and Chronic Health Evaluation, SOFA: Sequential Organ Failure Assessment, EASIX: Endothelial Activation and Stress Index, log2-EASIX: Log2-transformed Endothelial Activation and Stress Index. Categorical variables are presented as *n* (%), with percentages calculated using the corresponding column total as the denominator.

**Table 3:** Discriminative performance of log2-EASIX for in-hospital mortality assessed by ROC analysis.

Variables	AUC (95% CI)	Cut-Off Value	Sensitivity (95% CI)	Specificity (95% CI)	PPV (95% CI)	NPV (95% CI)	Youden Index	*p*-Value
log2-EASIX	0.673 (0.648–0.698)	1.47 (raw EASIX: 2.77)	0.59 (0.56–0.62)	0.70 (0.67–0.73)	0.71 (0.68–0.74)	0.57 (0.54–0.60)	0.284	<0.001

log2-EASIX: Log2-transformed Endothelial Activation and Stress Index, PPV: Positive Predictive Value, NPV: Negative Predictive Value. ROC analysis was performed on the entire study cohort (*n* = 1787).

**Table 4:** Comparison of demographic characteristics, clinical outcomes, and severity-related parameters according to log2-EASIX groups.

Variables	High log2-EASIX Score (≥1.47) Mean ± SD, Median (Q1–Q3) or *n* (%) *n* = 824	Low log2-EASIX Score (<1.47) Mean ± SD, Median (Q1–Q3) or *n* (%) *n* = 963	*p*-Value
**Gender**			0.938
Female	363 (44.1)	426 (44.2)	
Male	461 (55.9)	537 (55.8)	
Age, years	68.00 (57.00–78.00)	69.00 (57.00–79.00)	0.091
In-hospital mortality	588 (71.4)	416 (43.2)	<0.001
IMV need within 24 h	733 (89.0)	843 (87.5)	0.355
CRRT need within 7 d	396 (48.1)	168 (17.4)	<0.001
VP need within 24 h	791 (96.0)	871 (90.4)	<0.001
Second VP need	335 (40.7)	216 (22.4)	<0.001
≥1 comorbidity	225 (27.3)	141 (14.6)	<0.001
LOS ICU, days	5.88 (1.54–13.89)	7.29 (3.46–13.84)	<0.001
Lactate, mmol/L	2.40 (1.35–4.50)	1.75 (1.23–2.50)	<0.001
Albumin, g/L	26.95 ± 6.59	28.07 ± 6.53	<0.001
APACHE II	32.00 (26.00–36.50)	26.00 (22.00–32.00)	<0.001
SOFA	12.00 (8.00–14.00)	9.00 (6.00–12.00)	<0.001

IMV: Invasive Mechanical Ventilation, CRRT: Continuous Renal Replacement Therapy, VP: Vasopressor, LOS ICU: Length of Intensive Care Unit Stay, APACHE II: Acute Physiology and Chronic Health Evaluation, SOFA: Sequential Organ Failure Assessment, log2-EASIX: Log2-transformed Endothelial Activation and Stress Index. Categorical variables are presented as *n* (%), with percentages calculated using the corresponding column total as the denominator.

**Table 5:** Univariable logistic regression analysis of factors associated with in-hospital mortality.

Variables	OR (95% CI)	β Coefficient	*p*-Value
Age, years	1.011 (1.005–1.017)	0.011	<0.001
Gender	0.788 (0.653–0.952)	−0.238	0.014
IMV need within 24 h	0.725 (0.539–0.975)	−0.322	0.033
CRRT need within 7 d	2.099 (1.702–2.589)	0.741	<0.001
VP need within 24 h	7.619 (4.677–12.414)	2.031	<0.001
Second VP need	15.785 (11.465–21.733)	2.759	<0.001
≥1 comorbidity	1.315 (1.040–1.664)	0.274	0.022
LOS ICU, days	0.959 (0.949–0.969)	−0.042	<0.001
pH	0.103 (0.054–0.195)	−2.276	<0.001
BE, mmol/L	0.937 (0.925–0.949)	−0.065	<0.001
HCO_3_, mmol/L	0.924 (0.908–0.939)	−0.079	<0.001
PaCO_2_, mmHg	0.994 (0.989–1.000)	−0.006	0.046
Lactate, mmol/L	1.275 (1.220–1.332)	0.243	<0.001
Urea, mg/dL	1.006 (1.004–1.007)	0.006	<0.001
Creatinine, mg/dL	1.136 (1.075–1.200)	0.128	<0.001
Total bilirubin, mg/dL	1.312 (1.210–1.424)	0.272	<0.001
CRP, mg/L	1.001 (1.000–1.001)	0.001	0.137
PCT, ng/mL	1.007 (1.003–1.011)	0.007	0.001
Hgb, g/dL	0.878 (0.842–0.915)	−0.130	<0.001
PLT × 10^9^/L	0.998 (0.997–0.998)	−0.002	<0.001
Albumin, g/L	0.944 (0.930–0.958)	−0.058	<0.001
Pro-BNP, pg/mL	1.000 (1.000–1.000)	0.000	<0.001
LDH, U/L	1.001 (1.000–1.001)	0.001	<0.001
D-dimer, mg/L	1.059 (1.031–1.087)	0.057	<0.001
INR	2.231 (1.812–2.747)	0.803	<0.001
Fibrinogen, mg/dL	0.999 (0.998–0.999)	−0.001	<0.001
SpO_2_, %	0.963 (0.948–0.978)	−0.038	<0.001
HR, beats/min	1.007 (1.002–1.011)	0.007	0.002
APACHE II	1.119 (1.103–1.135)	0.112	<0.001
SOFA	1.318 (1.275–1.362)	0.276	<0.001
log2-EASIX	1.373 (1.302–1.447)	0.317	<0.001
High log2-EASIX (≥1.47)	3.276 (2.689–3.992)	1.187	<0.001

IMV: Invasive Mechanical Ventilation, CRRT: Continuous Renal Replacement Therapy, VP: Vasopressor, LOS ICU: Length of Intensive Care Unit Stay, BE: Base Excess, PaCO_2_: Partial Pressure of Carbon Dioxide, CRP: C-Reactive Protein, PCT: Procalcitonin, Hgb: Hemoglobin, PLT: Platelet Count, Pro-BNP: Pro-B-type Natriuretic Peptide, LDH: Lactate Dehydrogenase, INR: International Normalized Ratio, SpO_2_: Peripheral Oxygen Saturation, HR: Heart Rate, APACHE II: Acute Physiology and Chronic Health Evaluation, SOFA: Sequential Organ Failure Assessment, log2-EASIX: Log2-transformed Endothelial Activation and Stress Index. The number of patients included in each univariable analysis varied depending on the availability of data for the variable.

**Table 6:** Multivariable logistic regression models evaluating associations with in-hospital mortality.

Variables	Model 1			Model 2			Model 3		
	OR (95% CI)	β Coefficient	*p*-Value	OR (95% CI)	β Coefficient	*p*-Value	OR (95% CI)	β Coefficient	*p*-Value
Age	1.012 (1.006–1.018)	0.012	<0.001	1.001 (0.994–1.008)	0.001	0.814	1.001 (0.994–1.008)	0.001	0.807
Gender	0.817 (0.664–1.005)	−0.203	0.055	0.748 (0.596–0.940)	−0.290	0.013	0.748 (0.595–0.939)	−0.291	0.012
≥1 comorbidity	1.123 (0.864–1.458)	0.116	0.385	1.172 (0.877–1.568)	0.159	0.284	1.170 (0.874–1.565)	0.157	0.292
Albumin	0.951 (0.935–0.966)	−0.050	<0.001	0.947 (0.931–0.964)	−0.054	<0.001	0.949 (0.932–0.966)	−0.053	<0.001
Lactate	1.199 (1.146–1.255)	0.182	<0.001	1.140 (1.084–1.199)	0.131	<0.001	1.150 (1.094–1.208)	0.139	<0.001
APACHE II				1.108 (1.091–1.125)	0.102	<0.001	1.107 (1.091–1.124)	0.102	<0.001
log2-EASIX score	1.275 (1.203–1.350)	0.243	<0.001	1.191 (1.119–1.268)	0.175	<0.001			
High log2-EASIX (≥1.47)							2.039 (1.610–2.584)	0.713	<0.001

Model 1: Clinical model, Model 2: Severity-adjusted model, Model 3: Severity-adjusted model using the ROC-derived EASIX cut-off. Model 1 was adjusted for age, sex, presence of at least one comorbidity, albumin, lactate, and log2-transformed EASIX. Model 2 additionally included the APACHE II score to adjust for disease severity while retaining log2-transformed EASIX as a continuous variable. Model 3 was identical to Model 2, except that log2-transformed EASIX was replaced with a dichotomized EASIX variable (high EASIX: ≥1.47 vs. low EASIX: <1.47). Multicollinearity was assessed using the variance inflation factor (VIF) and tolerance statistics, with no evidence of multicollinearity (all VIF values < 2.0 and tolerance values > 0.80). Model performance was evaluated using the Omnibus test, Nagelkerke R^2^, and the Hosmer–Lemeshow goodness-of-fit test. All three models were statistically significant (Omnibus test, *p* < 0.001). The Hosmer–Lemeshow test indicated acceptable calibration for Models 2 and 3 (*p* = 0.065 and *p* = 0.095, respectively), while the Hosmer–Lemeshow test for Model 1 was statistically significant (*p* = 0.040), indicating evidence of lack of fit. Nagelkerke R^2^ values were 0.221, 0.320, and 0.323 for Models 1, 2, and 3, respectively. APACHE II: Acute Physiology and Chronic Health Evaluation, log2-EASIX: Log2-transformed Endothelial Activation and Stress Index. The numbers of patients with complete data included in Models 1, 2, and 3 were 1759, 1608, and 1608, respectively.

**Table 7:** Comparison of the discriminative performance of log2-EASIX, APACHE II, and the combined model for in-hospital mortality.

Variables	AUC (95% CI)	Nagelkerke R^2^	Hosmer-Lemeshow *p*-Value	Omnibus *p*-Value	*p*-Value
log2-EASIX	0.673 (0.648–0.698)	0.118	0.442	<0.001	<0.001
APACHE II	0.740 (0.716–0.764)	0.230	0.648	<0.001	<0.001
log2-EASIX + APACHE II	0.761 (0.738–0.784)	0.267	0.224	<0.001	<0.001

DeLong test demonstrated that the AUC of the log2-EASIX + APACHE II model was significantly higher than that of the APACHE II model alone (ΔAUC = 0.021, 95% CI: 0.010–0.032; *p* < 0.001). log2-EASIX: Log2-transformed Endothelial Activation and Stress Index. APACHE II: Acute Physiology and Chronic Health Evaluation. The log2-EASIX model included 1787 patients, whereas the APACHE II and the combined APACHE II + log2-EASIX models included 1630 patients with complete APACHE II data. The DeLong comparison between APACHE II and the combined model was conducted in these 1630 patients.

**Table 8:** Univariable and multivariable logistic regression analyses for secondary clinical outcomes.

Variables	Univariate			Multivariate		
	OR (95% CI)	β Coefficient	*p*-Value	OR (95% CI)	β Coefficient	*p*-Value
IMV need within 24 h						
Age	0.994 (0.985–1.003)	−0.006	0.186			
Gender	1.408 (1.056–1.878)	0.342	0.020	1.423 (1.051–1.925)	0.352	0.022
≥1 comorbidity	0.858 (0.608–1.212)	−0.153	0.385			
pH	0.004 (0.001–0.015)	−5.414	<0.001	0.025 (0.005–0.114)	−3.703	<0.001
PaCO_2_	1.039 (1.026–1.051)	0.038	<0.001	1.027 (1.011–1.042)	0.026	<0.001
SpO_2_	0.977 (0.954–1.001)	−0.023	0.061			
Lactate	1.125 (1.060–1.194)	0.118	<0.001	1.044 (0.974–1.119)	0.043	0.227
APACHE II	1.036 (1.019–1.053)	0.035	<0.001	1.033 (1.015–1.052)	0.033	<0.001
SOFA	1.123 (1.082–1.165)	0.116	<0.001			
log2-EASIX	1.074 (1.001–1.152)	0.071	0.047	0.961 (0.888–1.041)	−0.039	0.334
VP need within 24 h						
Age	1.018 (1.007–1.028)	0.017	<0.001			
Gender	0.993 (0.689–1.433)	−0.007	0.972			
≥1 comorbidity	0.765 (0.501–1.169)	−0.267	0.216			
pH	0.084 (0.022–0.314)	−2.481	<0.001	0.357 (0.068–1.871)	−1.029	0.223
Lactate	1.431 (1.244–1.645)	0.358	<0.001	1.294 (1.108–1.511)	0.258	0.001
BE	0.952 (0.930–0.975)	−0.049	<0.001			
HR	1.006 (0.997–1.015)	0.006	0.210			
APACHE II	1.107 (1.081–1.133)	0.102	<0.001	1.098 (1.071–1.125)	0.094	<0.001
SOFA	1.395 (1.307–1.489)	0.333	<0.001			
log2-EASIX	1.233 (1.117–1.360)	0.209	<0.001	1.036 (0.925–1.161)	0.036	0.541
Second VP need						
Age	0.999 (0.993–1.005)	−0.001	0.776			
Gender	0.941 (0.769–1.152)	−0.061	0.555			
≥1 comorbidity	0.879 (0.683–1.132)	−0.128	0.319			
pH	0.074 (0.039–0.143)	−2.602	<0.001	0.726 (0.307–1.717)	−0.321	0.466
Lactate	1.201 (1.164–1.239)	0.183	<0.001	1.132 (1.087–1.178)	0.124	<0.001
BE	0.936 (0.923–0.948)	−0.066	<0.001			
HR	1.004 (0.999–1.008)	0.004	0.115			
APACHE II	1.078 (1.063–1.093)	0.075	<0.001	1.062 (1.047–1.078)	0.060	<0.001
SOFA	1.210 (1.172–1.250)	0.191	<0.001			
log2-EASIX	1.262 (1.202–1.325)	0.233	<0.001	1.104 (1.040–1.171)	0.099	0.001
CRRT need within 7 d						
Age	0.993 (0.987–0.999)	−0.007	0.025			
Gender	0.939 (0.768–1.148)	−0.063	0.540			
≥1 comorbidity	2.978 (2.351–3.773)	1.091	<0.001	2.344 (1.782–3.083)	0.852	<0.001
pH	0.096 (0.050–0.184)	−2.339	<0.001			
BE	0.934 (0.922–0.947)	−0.068	<0.001			
HCO_3_	0.912 (0.896–0.929)	−0.092	<0.001			
Lactate	0.996 (0.970–1.024)	−0.004	0.797			
Urea	1.011 (1.009–1.013)	0.011	<0.001	1.007 (1.005–1.009)	0.007	<0.001
APACHE II	1.037 (1.024–1.050)	0.036	<0.001	1.013 (0.999–1.027)	0.013	0.062
SOFA	1.125 (1.095–1.156)	0.118	<0.001			
log2-EASIX	1.365 (1.297–1.437)	0.311	<0.001	1.317 (1.236–1.404)	0.276	<0.001

IMV: Invasive Mechanical Ventilation, PaCO_2_: Partial Pressure of Carbon Dioxide, SpO_2_: Peripheral Oxygen Saturation, APACHE II: Acute Physiology and Chronic Health Evaluation, SOFA: Sequential Organ Failure Assessment, log2- EASIX: Log2-transformed Endothelial Activation and Stress Index, VP: Vasopressor, BE: Base Excess, HR: Heart Rate, CRRT: Continuous Renal Replacement Therapy. Blank multivariable cells indicate variables excluded in the final adjusted model. Female sex was used as the reference category. The numbers of patients with complete data included in the multivariable models were 1625 for IMV requirement, 1630 for vasopressor requirement, 1630 for second-line vasopressor requirement, and 1630 for CRRT requirement.

**Table 9:** Discriminative performance of log2-EASIX for secondary clinical outcomes assessed by ROC analysis.

Variables	AUC (95% CI)	Cut-Off Value	Sensitivity	Specificity	Youden Index	*p*-Value
IMV need within 24 h	0.539 (0.497–0.581)	0.55	0.67	0.43	0.083	0.066
VP need within 24 h	0.618 (0.570–0.666)	1.46	0.48	0.74	0.213	<0.001
Second VP need	0.641 (0.614–0.669)	1.89	0.54	0.68	0.220	<0.001
CRRT need within 7 d	0.712 (0.687–0.736)	1.41	0.72	0.64	0.364	<0.001

IMV: Invasive Mechanical Ventilation, VP: Vasopressor, CRRT: Continuous Renal Replacement Therapy. ROC analyses were performed on the entire study cohort (*n* = 1787).

## Data Availability

The datasets used and/or analyzed during the current study are available from the corresponding author on reasonable request. The data are not publicly available due to ethical restrictions and the need to protect patient privacy.

## Supplementary Materials

The following supporting information can be downloaded at: https://www.mdpi.com/article/10.3390/life16091429/s1, Table S1: Detailed laboratory characteristics according to in-hospital mortality. Table S2: Detailed laboratory characteristics according to log2-EASIX groups.

## Abbreviations

ICU	Intensive Care Unit
IMV	Invasive Mechanical Ventilation
CRRT	Continuous Renal Replacement Therapy
EASIX	Endothelial Activation and Stress Index
LDH	Lactate Dehydrogenase
PLT	Platelet Count
COVID-19	Coronavirus Disease-2019
APACHE II	Acute Physiology and Chronic Health Evaluation
HR	Heart Rate
SpO_2_	Partial Oxygen Pressure
WBC	White Blood Cell Count
Hgb	Hemoglobin
Pro-BNP	Pro-B-Type Natriuretic Peptide
PaO_2_	Partial Oxygen Pressure
PaCO_2_	Partial Carbon Dioxide Pressure
BE	Base Excess
HCO_3_	Bicarbonate
INR	International Normalized Ratio
CRP	C-Reactive Protein
PCT	Procalcitonin
ROC	Receiver Operating Characteristic
SOFA	Sequential Organ Failure Assessment
MAP	Mean Arterial Pressure
SD	Standard Deviation
IQR	Interquartile Range
VIF	Variance Inflation Factor
AUC	Area Under Curve
CI	Confidence Interval
LOS	Length of Stay
PPV	Positive Predictive Value
NPV	Negative Predictive Value
Ang-2	Angiopoietin-2
IGF-1	Insulin-like Growth Factor
