# Supplementary material for: Association of the Endothelial Activation and Stress Index (EASIX) with Clinical Outcomes in Patients with Pneumosepsis: A Retrospective Cohort Study
Source: Life (Basel). 2026 Aug 28;16(9):1429. doi: 10.3390/life16091429 (PMC13608568; doi:10.3390/life16091429)
Supplement: Supplementary file 1 [file life-16-01429-s001.zip › life-4466720-supplementary.pdf]

Supplementary Table S1. Detailed laboratory characteristics according to in-hospital mortality

**Variables**	**Non-survivors Mean±SD, Median (Q1-Q3) or n (%) *n*=1004**	**Survivors Mean±SD, Median (Q1-Q3) or n (%) *n*=783**	***p*-value**
pH	7.31 (7.21-7.39)	7.32 (7.23-7.40)	<0.001
BE, mmol/L	-6.76±8.23	-2.76±7.47	<0.001
HCO_3, mmol/L	19.12±5.97	22.13±5.97	<0.001
PaO_2, mmHg	89.70 (60.28-130.00)	94.45 (67.80-136.75)	0.180
$PaCO_2$, mmHg	40.70 (34.60-49.30)	42.15 (35.53-52.45)	0.003
Urea, mg/dL	85.70 (51.40-128.70)	62.60 (38.65-100.05)	<0.001
Creatinine, mg/dL	1.64 (0.87-2.77)	1.30 (0.77-2.23)	<0.001
Total bilirubin, mg/dL	0.69 (0.43-1.75)	0.58 (0.34-1.06)	<0.001
CRP, mg/L	124.65 (63.40-212.00)	103.00 (46.35-200.25)	0.023
PCT, ng/mL	3.33 (0.88-18.71)	1.95 (0.52-10.58)	<0.001
WBC$\times10^9$/L	13.18 (8.01-18.48)	11.85 (7.91-18.55)	0.888
Hgb, g/dL	10.00 (8.20-11.40)	11.10 (9.40-12.63)	<0.001
PLT$\times10^9$/L	214.10 (119.25-302.00)	249.00 (173-330.75)	<0.001
Pro-BNP, pg/mL	7388.00 (2718.00-24504.00)	4225.50 (841.33-11620.50)	<0.001
LDH, U/L	352.50 (237.75-620.00)	285.50 (215.00-424.75)	<0.001
D-dimer, mg/L	3.42 (1.76-7.24)	2.82 (1.25-6.20)	<0.001
INR	1.39 (1.22-1.77)	1.26 (1.14-1.44)	<0.001
Fibrinogen, mg/dL	420.96 (297.00-569.62)	482.99 (352.81-624.00)	<0.001
SpO_2, %	95.00 (91.00-99.00)	95.00 (90.88-98.93)	<0.001
HR, beats/min	102.33 (84.33-117.33)	98.17 (83.50-121.17)	0.007

BE: Base Excess, PaO_2: Partial Pressure of Oxygen, $PaCO_2$: Partial Pressure of Carbon Dioxide, CRP: C-Reactive Protein, PCT: Procalcitonin, WBC: White Blood Cell, Hgb: Hemoglobin, PLT: Platelet Count, Pro-BNP: Pro-B-type Natriuretic Peptide, LDH: Lactate Dehydrogenase, INR: International Normalized Ratio, SpO_2: Peripheral Oxygen Saturation, HR: Heart Rate.

Supplementary Table S2. Detailed laboratory characteristics according to log2-EASIX groups.

**Variables**	**High log2-EASIX Score (≥1.47) Mean±SD, Median (Q1-Q3) or n (%) *n*=824**	**Low log2-EASIX Score (<1.47) Mean±SD, Median (Q1-Q3) or n (%) *n*=963**	***p*-value**
pH	7.27 (7.19-7.39)	7.34 (7.26-7.41)	<0.001
BE, mmol/L	-6.70 (-11.55 – -2.40)	-1.70 (-6.10–1.90)	<0.001
HCO_3, mmol/L	18.90 (15.50-22.25)	22.75 (18.83-25.90)	<0.001
PaO_2, mmHg	92.10 (62.10-131.25)	91.90 (62.90-134.00)	0.455
$PaCO_2$, mmHg	40.60 (33.60-49.45)	42.90 (36.55-53.25)	<0.001

Urea, mg/dL	99.40 (64.25-141.50)	53.55 (34.43-82.00)	<0.001
Creatinine, mg/dL	2.51 (1.52-3.93)	0.95 (0.62-1.49)	<0.001
Total bilirubin, mg/dL	0.69 (0.41-1.73)	0.59 (0.36-0.98)	<0.001
CRP, mg/L	127.00 (64.50-214.45)	103.00 (42.75-190.50)	<0.001
PCT, ng/mL	2.88 (0.92-35.08)	0.89 (0.27-4.43)	<0.001
WBC $\times 10^9$/L	11.45 (7.01-17.62)	12.94 (8.77-19.40)	0.140
Hgb, g/dL	9.70 (8.00-11.60)	11.20 (9.50-12.50)	<0.001
PLT $\times 10^9$/L	161.00 (86.00-222.50)	277.00 (207.00-367.25)	<0.001
Pro-BNP, pg/mL	11935.00 (3467.50-30686.50)	3194.00 (843.53-8202.25)	<0.001
LDH, U/L	417.00 (286.50-712.50)	251.50 (188.25-322.50)	<0.001
D-dimer, mg/L	4.00 (1.79-8.62)	2.72 (1.21-5.37)	<0.001
INR	1.41 (1.21-1.71)	1.26 (1.14-1.43)	<0.001
Fibrinogen, mg/dL	419.00 (276.75-581.25)	478.50 (354.82-613.00)	<0.001
SpO_2, %	95.00 (91.00-98.50)	95.00 (90.83-99.00)	0.005
HR, beats/min	104.00 (84.17-120.33)	97.33 (83.17-119.00)	0.174

BE: Base Excess, PaO_2: Partial Pressure of Oxygen, $PaCO_2$: Partial Pressure of Carbon Dioxide, CRP: C-Reactive Protein, PCT: Procalcitonin, WBC: White Blood Cell, Hgb: Hemoglobin, PLT: Platelet Count, Pro-BNP: Pro-B-type Natriuretic Peptide, LDH: Lactate Dehydrogenase, INR: International Normalized Ratio, SpO_2: Peripheral Oxygen Saturation, HR: Heart Rate, log2-EASIX: Log2-transformed Endothelial Activation and Stress Index.
